# Age-related sex differences in factors associated with suicidal ideation: a short-term longitudinal study

**DOI:** 10.3389/fpsyg.2026.1813220

**Published:** 2026-08-19

**Authors:** Ayumi Fusejima, Azusa Arimoto, Kenkichi Takase, Tomoyuki Miyazaki

**Affiliations:** 1 College of Media Information, Kanazawa Institute of Technology, Nonoichi, Ishikawa, Japan; 2 Faculty of Medicine, Yokohama City University, Yokohama, Kanagawa, Japan; 3 Faculty of Letters, Chuo University, Hachioji, Tokyo, Japan; 4 Center for Promotion of Research and Industry-Academic Collaboration, Yokohama City University, Yokohama, Kanagawa, Japan

**Keywords:** depressive symptoms, latent change model, perceived social support, suicidal ideation, well-being

## Abstract

**Objective:**

This study examines longitudinal associations among suicidal ideation, perceived social support, well-being, and depressive symptoms, and compares them across age groups and sex.

**Methods:**

Participants (*N* = 1,078; aged 15–49 years) completed online questionnaires in January 2024 (T1) and February 2024 (T2), over a one-month interval. Suicidal ideation was assessed using the short form of the Scale for Suicide Ideation, perceived social support using the Multidimensional Scale of Perceived Social Support, depressive symptoms using the Patient Health Questionnaire-9, and well-being using the PERMA-Profiler. Using a latent change model, we estimated baseline levels (“Level”) and latent T1–T2 changes (“Change”) in suicidal ideation, mental health (well-being and depressive symptoms), and perceived social support. Here, “change in suicidal ideation” refers to latent short-term change from T1 to T2 and may reflect emergence, worsening, or improvement. Participants were divided into four groups by age and sex (younger women, younger men, middle-aged women, and middle-aged men), and multi-group analyses compared associations across groups. Younger and middle-aged participants were aged 15–29 and 30–49 years, respectively.

**Results:**

Associations among the latent change factors (i.e., Change–Change correlations) differed by group. Among younger women, worsening suicidal ideation covaried with worsening depressive symptoms (*r* = 0.62, *p* < 0.001) and declines in well-being (*r* = −0.59, *p* < 0.001) and perceived social support (*r* = −0.37, *p* < 0.001). In contrast, Change–Change correlations among younger men were not significant. Among middle-aged women, worsening suicidal ideation covaried with declines in perceived social support (*r* = −0.21, *p* = 0.026) and well-being (*r* = −0.21, *p* = 0.023). Among middle-aged men, worsening suicidal ideation covaried with worsening depressive symptoms (*r* = 0.41, *p* < 0.001) and declines in perceived social support (*r* = −0.29, *p* = 0.003).

**Discussion:**

Overall, perceived social support was associated with lower short-term change in suicidal ideation in all groups except younger men, suggesting that social support may be a relevant correlate of short-term co-change in suicidal ideation. Particularly among younger women, short-term changes in well-being and depressive symptoms may be especially important correlates of suicidal ideation, whereas correlates of short-term change in suicidal ideation may differ among younger men.

## 1 Introduction

Many countries continue to report a high prevalence of suicidal ideation among youth. Across 149 regions worldwide, lifetime and past-year prevalence of suicidal ideation among youth younger than 22 years were 20.5 and 15.1%, respectively, based on reports published between 1981 and 2021 (Van Meter et al., 2023). In the United States, the proportion of female high school students who seriously considered suicide increased from 24.1% in 2019 to 30% in 2021 (Gaylor et al., 2023). In Japan, the past-year prevalence of suicidal ideation among youth has been reported to range from 5.7 to 21.7%. However, estimates vary depending on the survey and timing (Ministry of Health, Labour and Welfare, 2021; Nippon Foundation, 2019; Nippon Foundation, 2021).

Notably, suicidal ideation prevalence is higher among females than males across many age groups (Boyd et al., 2015). In Japan, similarly, females report suicidal ideation more frequently than males (Nippon Foundation, 2019; Nippon Foundation, 2021). Moreover, past-year prevalence of suicidal ideation and attempts among junior and senior high school students is higher among girls than boys (Nagamitsu et al., 2020). Additionally, suicidal ideation prevalence is higher during late adolescence (15–19 years) and follows an age gradient, declining progressively through young adulthood and into middle and older adulthood (Nippon Foundation, 2021). Age patterns, however, may not be uniform across countries. Taken together, these findings suggest that sex and age differences in suicidal ideation may vary across cultural contexts, underscoring the need for further research that explicitly examines these differences (Kim et al., 2023; Nippon Foundation, 2021). In Western settings, age- and sex-related patterns of suicidal ideation have also been documented, but these patterns are not consistent across countries. European studies have reported that women tend to report suicidal ideation more frequently than men, while age-related patterns vary substantially across countries and are not always linear in middle and older adulthood (Boyd et al., 2015; Cabello et al., 2021). In Japan, the high number of suicides among school-aged children and the recent increase among younger women are major public health concerns (Ministry of Health, Labour and Welfare, 2025), underscoring the need to identify risk factors and develop age- and sex-sensitive countermeasures.

At the same time, this issue should not be viewed only in relation to youth. In Japan, suicide mortality remains substantially higher among men overall, while recent national statistics also indicate persistent concern in specific age and sex groups, including children and young women (Ministry of Health, Labour and Welfare, 2025). In addition, previous research has shown that psychosocial correlates of suicidal ideation may differ by sex among middle-aged adults (Sugawara et al., 2013). These findings suggest that age- and sex-related patterns of suicide risk should be examined not only in youth but also across adult life stages, particularly in the Japanese context, where mortality patterns and psychosocial correlates do not necessarily parallel those reported in Western countries. These findings further support the need to examine age- and sex-specific patterns within Japan rather than inferring them from other cultural contexts.

In addition, the present age grouping was chosen for both substantive and practical reasons. In Japan, suicidal ideation has been reported to be particularly high in late adolescence and to decline across adulthood, making it meaningful to distinguish younger adults from middle-aged adults in the present analyses (Nippon Foundation, 2021). At the same time, age patterns may not be uniform across countries; for example, suicidal ideation has been reported to increase with age except among those in their 20s in Korea (Kim et al., 2023). These findings suggest that age- and sex-related patterns of suicidal ideation may vary across cultural contexts and underscore the need for culturally specific evidence. Furthermore, dividing the sample into four age-by-sex groups (younger women, younger men, middle-aged women, and middle-aged men) provided an analytically feasible framework while retaining adequate group sizes for comparison. Accordingly, this study defines younger adults and middle-aged adults as those aged 15–29 and 30–49 years, respectively. In this study, *sex* refers to the binary female/male category recorded in the survey dataset (self-reported by participants). Accordingly, *sex differences* indicate differences between these two categories and do not capture the full spectrum of sex- or gender-related diversity.

Although not all individuals with suicidal ideation attempt suicide, persistent suicidal ideation can cause significant psychological distress (Troister et al., 2013). Suicidal ideation is a risk factor for suicide (Brown et al., 2000), and persistent ideation, over weeks to months, predicts subsequent suicidal behavior (Anestis et al., 2014). Furthermore, midlife onward, while overall levels of suicidal ideation between men and women may differ insignificantly, sex differences emerge in associated psychosocial factors (Lu et al., 2020; Sugawara et al., 2013).

Examining short-term longitudinal changes in suicidal ideation by age and sex may help identify factors associated with increases or decreases in suicidal ideation and may inform age- and sex-sensitive approaches to suicide prevention. This study examines mental health (depressive symptoms and well-being) and social support as variables associated with longitudinal changes in suicidal ideation.

### 1.1 Two-factor model of mental health and suicidal ideation

Consistent with the two-factor model of mental health (Keyes and Lopez, 2002), this study conceptualizes depressive symptoms and well-being as negative and positive indicators of mental health. The association between depression and suicide risk has long been recognized. Mental disorders such as depression are key risk factors for suicide (World Health Organization, 2014), and depressive states are associated with suicidal behavior (Klonsky et al., 2016). However, psychopathology severity among young adult women (aged 18–24 years) does not necessarily predict suicidal ideation trajectory, whereas social support and positive mental health predict its remission (Teismann et al., 2016). Therefore, the extent to which depressive symptoms relate to suicidal ideation across age and sex remains unclear. Regarding sex differences in depression, the gap emerges but peaks in adolescence and narrows in adulthood (Salk et al., 2017). Sex differences have also been reported in the strength of the association between depression and suicidal ideation: among high school students (aged 15–18 years) and youth (aged 15–25 years), females are more likely to report suicidal ideation when depressive symptoms are at a moderate level than males; depression is a primary predictor of suicidal ideation among females, whereas factors such as age may play a relatively larger role among males (Allison et al., 2001; Ibrahim et al., 2017). Overall, the strength of the association between depressive symptoms and suicidal ideation and whether age-specific sex differences exist in this association remain unclear.

Similar to depressive symptoms, well-being has also been linked to suicide risk. Although definitions of well-being vary, it is commonly defined as a positive evaluation of one’s life, including positive emotions, engagement, satisfaction, and values (Diener and Seligman, 2004), and is often conceptualized as cognitive and affective self-evaluations of happiness. Another approach conceptualizes well-being as positive self-evaluations across five components, including subjective feelings, meaning, and accomplishment (Seligman, 2011). Higher comprehensive well-being that includes these components may help reduce suicide risk (Canter et al., 2024; Fusejima et al., 2022). Regarding sex differences in well-being, overall differences during childhood and adolescence are small. However, boys report slightly higher life satisfaction than girls (Brisson et al., 2023, 2025; Chen et al., 2020), suggesting potential sex differences in well-being. Life satisfaction, a component of well-being, is an independent predictor of suicidal ideation among females but not males (Hossain et al., 2016; Lu et al., 2020). Therefore, research on well-being and suicide has not always accounted for the effects of depressive symptoms, which are strongly related to suicide risk, and sex differences remain underexamined.

Among youth, females may be relatively more likely to be disadvantaged regarding suicidal ideation and the negative aspect of mental health (depressive symptoms) and potentially the positive aspect (well-being). Therefore, it is necessary to clarify age- and sex-specific associations between each dimension of mental health and suicidal ideation within a two-factor framework.

### 1.2 Perceived social support and suicidal ideation

Social support is widely regarded as a protective factor that mitigates suicidal ideation. Social isolation—such as limited social networks and the absence of close sources of social support (e.g., being unmarried or living alone)—is a suicide risk factor (Trout, 1980). Low perceived social support (i.e., the perception that one can obtain support from others) is linked to poorer mental and physical health, with evidence suggesting either unidirectional (Thoits, 1995) or bidirectional associations (Näher et al., 2020). Therefore, higher perceived social support may be associated with lower suicidal ideation.

Particularly, during adolescence, social ties may weaken, making the family a core source of support and a key indicator for suicide prevention (Motillon-Toudic et al., 2022). In a longitudinal study of young adult women (aged 18–24 years), baseline perceived social support and positive mental health predicted remission of suicidal ideation approximately 1.5 years later, suggesting that protective factors may be more important for the course of suicidal ideation than psychopathology severity (Teismann et al., 2016). Among individuals who experienced mental health problems during adolescence, higher perceived social support was associated with lower suicidal ideation 1 year later even after controlling for baseline depressive symptoms and suicidal ideation (Scardera et al., 2020). Therefore, perceived social support may be independently associated with lower suicide risk among youth, even when mental health is taken into account.

Evidence also suggests sex differences in perceived social support and in its protective association with suicidal ideation. Although effect sizes are generally small, women report higher perceived social support than men (Hinz et al., 2025). Among young adults, the protective role of social support for mental health has been reported to be stronger among women than men (Johansen et al., 2021). The interpersonal theory of suicide posits that thwarted belongingness (i.e., the sense of not being part of a group and lacking connection to others) increases suicide risk (Joiner, 2005; Van Orden et al., 2010). Some studies also indicate that the associations between connectedness, mental health, and suicidal ideation differ by sex: among women, poorer mental health increases thwarted belongingness, and thwarted belongingness increases suicidal ideation, whereas these associations are not observed among men (Donker et al., 2014). These findings suggest that perceptions and feelings regarding connectedness with others may influence suicidal ideation, and that this association may be stronger among women.

### 1.3 Aims

In summary, evidence increasingly indicates that women are disadvantaged in terms of depressive symptoms and suicide-related indicators from adolescence through young adulthood, whereas in age groups from middle adulthood onward, sex differences in depressive symptoms diminish and the direction and magnitude of sex differences vary across studies (Salk et al., 2017). Moreover, how sex differences in well-being and social support may change by age remain underexamined. Particularly, in Japan, few studies have comprehensively examined, within a single study, how sex differences in suicidal ideation, the two-factor model of mental health (depressive symptoms and well-being), and perceived social support differ between young people and middle-aged adults.

This study examines mental health (depressive symptoms and well-being) and perceived social support as factors associated with suicidal ideation, investigates their longitudinal associations, and compares differences in these relationships by age and sex. To examine longitudinal relationships among multiple variables, analyses of panel survey data with at least two time points are required. Accordingly, we applied a latent change model (LCM; McArdle and Nesselroade, 1994). This approach was chosen because it allows individual differences in short-term change to be modeled explicitly, distinguishes baseline level from subsequent change, and enables direct comparison of change-related associations across age-by-sex groups. Given the two-wave design and the study aim of examining co-change rather than average population effects, the LCM was considered more suitable than methods such as generalized estimating equations, which are less informative for modeling latent individual change.

## 2 Methods

### 2.1 Procedure and participants

We invited 74,996 individuals aged 15–49 years, registered as monitors with Cross Marketing Inc., to participate in a questionnaire survey. Participants were recruited via an online survey company using age- and sex-stratified quota sampling to achieve approximately balanced group sizes. Survey invitations were distributed to all registered monitors in the eligible age range, and respondents participated on a first-come, first-served basis until the predetermined quotas were filled. The first (Time 1 [T1]) and second (Time 2 [T2]) surveys were conducted in early January 2024 and early February 2024, respectively. The sample sizes and mean ages at T1 were 222 younger women (20.01 ± 4.59 years), 204 younger men (21.99 ± 4.67 years), 335 middle-aged women (40.25 ± 5.67 years), and 317 middle-aged men (40.72 ± 5.74 years). Of the 74,996 individuals to whom invitations were sent, 1,078 completed both waves, representing 1.44% of all invitees. However, because recruitment was closed once the predetermined quotas were filled, this proportion should not be interpreted as a conventional response rate. A total of 1,525 respondents completed the Time 1 (T1) survey, and 1,078 of these respondents also completed the Time 2 (T2) survey, yielding a retention rate of 70.7% from T1 to T2. To examine potential attrition bias, we compared respondents who completed both waves with those who dropped out after T1. The main analyses were conducted using complete-case data from respondents who completed both waves; respondents who did not complete T2 were excluded, and no imputation was performed. No item-level data were missing among respondents who completed both waves.

This study was approved by the institutional Human Ethics Committee of the Kanazawa Institute of Technology (Approval Number: 2308017). Before participation, participants received study information and provided informed consent. Written informed consent (i.e., signed paper forms) was not obtained because this study was conducted as an anonymous online questionnaire. Instead, participants provided informed consent/assent electronically by selecting a mandatory consent checkbox before proceeding. For participants under 18 years of age, the institutional ethics committee approved a consent procedure that did not require written parental consent, given the study’s minimal risk, its anonymous design, and the impracticability of collecting signed parental consent in an online survey context. Given the potential for poor physical or mental conditions because of survey participation, support resources and helplines were provided at the end of the survey.

### 2.2 Measures

#### 2.2.1 Suicidal ideation

Suicidal ideation was assessed using the shortened version (Sueki, 2019) of the Japanese self-report adaptation (Otsuka et al., 1998) of the Scale for Suicide Ideation (Beck et al., 1979). The scale comprises six items rated on a three-point scale (0–2). Total scores represented suicidal ideation. Although item 9 of the PHQ-9 includes death- and self-harm-related thoughts, the suicidal ideation scale used in this study assessed a broader and more severe range of suicide-related cognitions, including desire to die, intensity and persistence of suicidal thoughts, suicidal intent, and consideration of methods.

#### 2.2.2 Perceived social support

Perceived social support was assessed using the Japanese version (Iwasa et al., 2007) of the Multidimensional Scale of Perceived Social Support (Zimet et al., 1988). The scale comprises 12 items rated on a seven-point scale and includes three subscales: support from significant other, family, and friends.

#### 2.2.3 Depressive symptoms

Depressive symptoms were assessed using the Japanese version (Muramatsu et al., 2018) of the Patient Health Questionnaire-9 (Kroenke et al., 2001), a nine-item scale rated on a four-point response format.

#### 2.2.4 Well-being

Well-being was assessed using the Japanese version (Shiotani et al., 2015) of the PERMA-Profiler (Butler and Kern, 2016). This scale assesses well-being multidimensionally across five components (positive emotion, engagement, relationships, meaning, and accomplishment). We used the 15 items (three items for each component) as the indicator of well-being.

#### 2.2.5 Internal consistency

In the present sample, all study measures showed high internal consistency. At T1, Cronbach’s *α* coefficients were α = 0.906 for suicidal ideation, α = 0.970 for perceived social support, α = 0.924 for depressive symptoms, and α = 0.970 for well-being.

In the present study, perceived social support refers to the perceived availability of support from specific interpersonal sources, namely significant others, family, and friends (Zimet et al., 1988). By contrast, well-being, as assessed with the PERMA-Profiler, reflects broader multidimensional positive functioning that includes positive emotion, engagement, relationships, meaning, and accomplishment (Butler and Kern, 2016). Thus, although the two constructs may partially overlap in interpersonal content, particularly through the relationships component, they are conceptually distinct: perceived social support captures access to supportive interpersonal resources, whereas well-being captures broader positive functioning across life domains.

### 2.3 Statistical analysis

**Figure 1:**
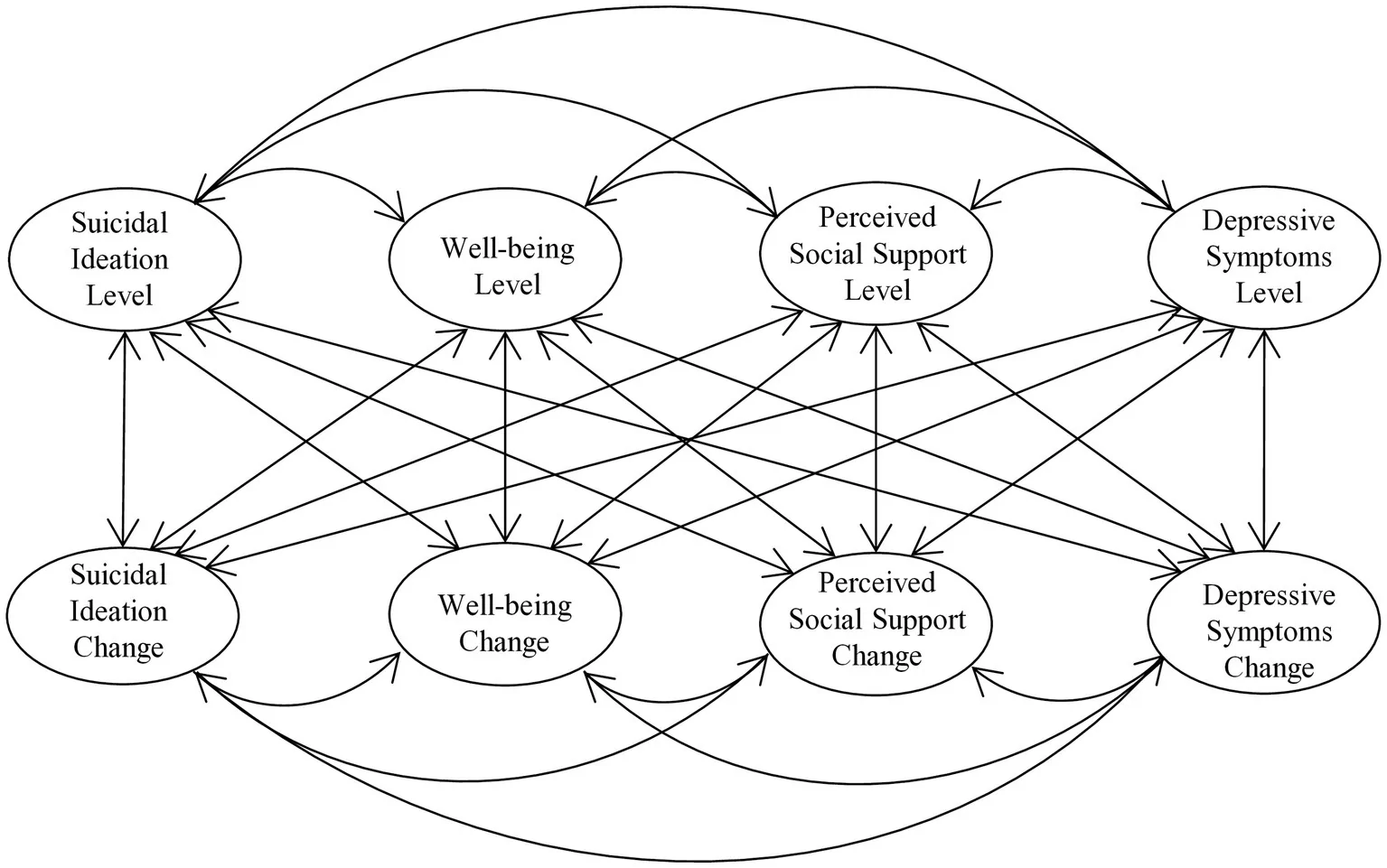
Parts of multiple latent change model. Age was also used in this model but is not shown in the figure.

Because the primary aim of this study was to examine short-term co-change among suicidal ideation, depressive symptoms, well-being, and perceived social support, we used a latent change model (LCM), which is well suited to separating baseline level from subsequent change and to estimating associations among latent change factors. We conducted a multi-group analysis across four groups (younger women, younger men, middle-aged women, and middle-aged men) using a latent change model (LCM; McArdle and Nesselroade, 1994). Following Takahashi (2017), we specified a hypothesized model including latent variables representing the initial level (“Level”) and the change from T1 to T2 (“Change”) for each construct (Figure 1). The factors at T1 and T2 were dummy factors with their means fixed at 0; the path coefficients from the latent variable representing Change to the T1 and T2 factors were fixed at 0 and 1, respectively. Covariances were specified between the error terms of the same items across T1 and T2. We further specified correlations between the latent variables representing Level and Change for each construct—social support, well-being, depressive symptoms, and suicidal ideation—by drawing bidirectional paths. Item-level and subscale-level observed variables were treated as continuous indicators in the SEM framework. Model parameters were estimated using maximum likelihood estimation. Because correlations among the Change factors in the latent change model provide insights relevant to suicide prevention interventions, we focused primarily on associations among Change factors. Because item 9 of the PHQ-9 assesses thoughts of being better off dead or self-harm, which may partially overlap with suicidal ideation, we conducted a sensitivity analysis excluding this item and re-estimated the main model.

To control for age effects, we also specified bidirectional paths between participants’ age at T1 and all study variables. Model fit was evaluated using the fit indices CFI, RMSEA, and SRMR.

To examine whether the constructs were measured equivalently across time and age-by-sex groups, we conducted longitudinal multigroup confirmatory factor analyses separately for suicidal ideation, depressive symptoms, well-being, and perceived social support. Item scores were used as indicators for suicidal ideation and depressive symptoms, whereas subscale scores were used as indicators for well-being and perceived social support. The four age-by-sex groups were analyzed simultaneously.

For each construct, we sequentially tested a configural model (Model 0); a longitudinal metric invariance model in which factor loadings were constrained to equality across T1 and T2 within each group (Model 1); and a longitudinal scalar invariance model in which indicator intercepts were additionally constrained to equality across T1 and T2 within each group (Model 2). We then tested between-group metric invariance by additionally constraining factor loadings to equality across the four groups (Model 3), followed by between-group scalar invariance by additionally constraining indicator intercepts to equality across the four groups (Model 4). Residual covariances were specified between the same indicators across T1 and T2.

Measurement invariance was evaluated using changes in CFI, RMSEA, and SRMR between successive models. Invariance was considered supported when the decrease in CFI did not exceed 0.010 and the increase in RMSEA did not exceed 0.015. Increases in SRMR of no more than 0.030 for metric invariance and 0.010 for scalar invariance were used as additional guidelines (Chen, 2007; Putnick and Bornstein, 2016).

We conducted a *post hoc* power analysis in G*Power (linear multiple regression, fixed model, *R*^2^ deviation from zero). With three predictors, an alpha level of 0.05, an effect size of *f*^2^ = 0.078, and a total sample size of 1,078, the achieved power was 1.00. Because post hoc power is a function of the observed effect size and sample size, it should be interpreted descriptively.

As a supplementary sensitivity analysis, we conducted group-specific linear regression analyses using observed change scores (T2–T1) in suicidal ideation as the dependent variable. Age (continuous) and observed changes in depressive symptoms, well-being, and perceived social support were entered simultaneously as predictors within each age-by-sex group.

To provide direct statistical tests of sex differences in the key Change–Change associations, we conducted supplementary nested multi-group comparisons separately within the younger and middle-aged groups. Specifically, two-group models were estimated for younger women versus younger men and for middle-aged women versus middle-aged men, and equality constraints were imposed on the focal correlations between change in suicidal ideation and changes in depressive symptoms, well-being, and perceived social support. Constrained and unconstrained models were compared using chi-square difference tests.

## 3 Results

### 3.1 Participant characteristics and descriptive statistics

Table 1 shows participant characteristics and descriptive statistics for each variable. Approximately half of the younger women and men were students, and approximately 40% were employed. Approximately 70 and 20% of the middle-aged women were employed and homemakers, respectively, whereas almost all middle-aged men were employed. Across all groups, approximately 10% were unemployed.

**Table 1:** Participant characteristics by age-sex group.

Variable	Younger women	Younger men	Middle-aged women	Middle-aged men
Employment status, *n* (%)				
Student	105 (47.3)	107 (52.5)	0 (0.0)	0 (0.0)
Employed	86 (38.7)	75 (36.8)	225 (67.2)	284 (89.6)
Homemaker	2 (0.9)	0 (0.0)	68 (20.3)	0 (0.0)
Unemployed	29 (13.1)	22 (10.8)	42 (12.5)	33 (10.4)
Marital status, *n* (%)				
Unmarried	201 (90.5)	192 (94.1)	138 (41.2)	195 (61.5)
Married	21 (9.5)	12 (5.9)	197 (58.8)	122 (38.5)
Having children, *n* (%)				
No	208 (93.7)	198 (97.1)	198 (59.1)	220 (69.4)
Yes	14 (6.3)	6 (2.9)	137 (40.9)	97 (30.6)
Perceived social support, mean (SD)	54.96 (20.51)	56.21 (18.31)	53.77 (19.40)	49.11 (19.51)
Depressive Symptoms, mean (SD)	7.83 (7.39)	4.89 (6.00)	6.82 (6.66)	5.83 (6.08)
Well-being, mean (SD)	4.90 (2.44)	5.72 (2.14)	4.89 (2.39)	4.68 (2.21)
Suicidal ideation, mean (SD)	9.06 (3.44)	8.04 (2.89)	8.50 (3.11)	8.03 (2.75)

Younger adults = 15–29 years; middle-aged adults = 30–49 years.

Regarding marital status, approximately 10% of both younger men and women were married, whereas 60 and 40% of middle-aged women and men were married. The proportion of participants with children was lowest among younger men (3%), followed by younger women (6%), middle-aged men (30%), and middle-aged women (approximately 40%).

Attrition analyses indicated that respondents who dropped out after T1 were younger and reported higher depressive symptoms at T1 than those who completed both waves. In contrast, no significant group differences were observed in sex distribution, T1 suicidal ideation, T1 well-being, or T1 perceived social support. Detailed comparisons between completers and non-completers are presented in Supplementary Table S1.

### 3.2 Evaluation of the hypothesized model

The hypothesized model yielded *χ*^2^(3904) = 8274.350 (*p* < 0.001). The fit indices indicated acceptable model fit (CFI = 0.920, RMSEA = 0.032, SRMR = 0.0617), suggesting that the overall model was adequate.

Factor loadings from the latent variables to the observed variables were significant and exceeded 0.59 in all groups. Regarding perceived social support, the factor loadings of the observed variables on the latent construct were largest for support from significant other (*β* = 0.96–0.99), followed by family support (*β* = 0.84–0.93), and friends support (*β* = 0.77–0.85). Regarding well-being, the factor loadings of the observed variables on the latent construct were positive emotion (*β* = 0.92–0.96, *p*s < 0.001), engagement (*β* = 0.86–0.90, *p*s < 0.001), relationships (*β* = 0.87–0.92, *p*s < 0.001), meaning (*β* = 0.89–0.94, *p*s < 0.001), and accomplishment (*β* = 0.83–0.92, *p*s < 0.001). No substantial differences in factor loadings were observed across the four groups. The variances and standard deviations of the latent variables representing Level and Change were significant, indicating that the latent variables were interpretable.

### 3.3 Measurement invariance

Longitudinal and between-group measurement invariance analyses were conducted separately for suicidal ideation, depressive symptoms, well-being, and perceived social support. The configural models showed acceptable fit for all four constructs. Imposing equality constraints on factor loadings and indicator intercepts across time and groups generally resulted in only small changes in CFI, RMSEA, and SRMR.

For suicidal ideation, depressive symptoms, and well-being, changes in the fit indices across all successive models were within the prespecified guidelines, supporting longitudinal and between-group metric and scalar invariance. For perceived social support, the changes in fit indices also supported longitudinal metric and scalar invariance and between-group metric invariance. When between-group equality constraints were additionally imposed on the indicator intercepts, the increase in RMSEA was 0.016, which was slightly larger than the guideline of 0.015. However, the corresponding changes in CFI (ΔCFI = −0.006) and SRMR (ΔSRMR = 0.001) were small, and the absolute fit of the scalar invariance model remained favorable (CFI = 0.993, RMSEA = 0.032, SRMR = 0.010).

Taken together, the results generally supported longitudinal and between-group measurement invariance for the four constructs, although the evidence for between-group scalar invariance of perceived social support was not unequivocal. Detailed results are presented in Supplementary Table S4.

Age was included as a covariate in the hypothesized model. No latent variables were significantly associated with age among younger women or middle-aged men. Among younger men, age was significantly associated with the Level of well-being (*r* = −0.36, *p* < 0.001) and the Level of perceived social support (*r* = −0.32, *p* < 0.001). Among middle-aged women, age was significantly associated with the Level of suicidal ideation (*r* = −0.14, *p* = 0.018).

### 3.4 Associations between level factors

Associations between Level factors were similar across age-by-sex groups (Tables 2, 3). Depressive symptoms, well-being, and perceived social support were significantly associated with suicidal ideation in all groups. The association between suicidal ideation and depressive symptoms was the strongest (*r*s = 0.74–0.83, *p*s < 0.001), whereas associations with well-being (*r*s = −0.57 to −0.67, *p*s < 0.001) and perceived social support (*r*s = −0.52 to −0.62, *p*s < 0.001) were of comparable magnitude. All other inter-variable associations were also significant; there were few group differences in the strength of these associations.

**Table 2:** Estimated correlations in “Level” and “Change” among younger adults.

Factor	Variable	Level				Change			
		(a)	(b)	(c)	(d)	(a)	(b)	(c)	(d)
Level	(a)		0.76^***^	−0.59^***^	−0.58^***^	−0.21^*^	−0.11	0.14	0.03
	(b)	0.79^***^		−0.72^***^	−0.65^***^	−0.05	−0.22^*^	0.23^*^	0.10
	(c)	−0.58^***^	−0.64^***^		0.79^***^	0.04	0.11	−0.39^***^	−0.10
	(d)	−0.52^***^	−0.57^***^	0.74^***^		−0.05	0.10	−0.21^*^	−0.07
Change	(a)	−0.30^**^	−0.17	0.09	0.05		0.53^***^	−0.39^***^	−0.37^***^
	(b)	−0.07	−0.18	0.01	−0.01	0.27^**^		−0.50^***^	−0.59^***^
	(c)	0.13	0.07	−0.20	−0.03	−0.08	−0.06		0.62^***^
	(d)	0.03	0.10	−0.20	−0.31	−0.02	−0.15	0.18	

(a) Perceived social support; (b) well-being; (c) depressive symptoms; (d) suicidal ideation. Correlations above the diagonal are for younger women; correlations below the diagonal are for younger men. ^*^p < 0.05, ^**^p < 0.01, ^***^p < 0.001.

**Table 3:** Estimated correlations in “Level” and “Change” among middle-aged adults.

Factor	Variable	Level				Change			
		(a)	(b)	(c)	(d)	(a)	(b)	(c)	(d)
Level	(a)		0.76^***^	−0.59^***^	−0.62^***^	−0.13	−0.11	0.11	−0.08
	(b)	0.74^***^		−0.75^***^	−0.67^***^	0.02	−0.27^***^	0.21^*^	−0.12
	(c)	−0.51^***^	−0.64^***^		0.83^***^	−0.09	0.00	−0.38^**^	0.15
	(d)	−0.53^***^	−0.59^***^	0.79^***^		−0.02	0.00	−0.18	−0.06
Change	(a)	−0.11	0.14^*^	0.01	0.03		0.33^***^	−0.14	−0.21^*^
	(b)	−0.08	−0.21^**^	0.06	0.03	0.34^***^		−0.26^***^	−0.21^*^
	(c)	0.03	−0.06	−0.38^***^	−0.16	−0.20^**^	−0.02		0.18
	(d)	0.11	0.01	−0.14	−0.26	−0.29^**^	−0.11	0.41^***^	

(a) Perceived social support; (b) well-being; (c) depressive symptoms; (d) suicidal ideation. Correlations above the diagonal are for middle-aged women; correlations below the diagonal are for middle-aged men. ^*^p < 0.05, ^**^p < 0.01, ^***^p < 0.001.

### 3.5 Associations between change factors

The associations between latent variables representing Change in suicidal ideation and Change in mental health and social support differed across age-by-sex groups. Among younger women, Change in suicidal ideation was significantly associated with Change in all other constructs. It was positively associated with depressive symptoms (*r* = 0.62, *p* < 0.001) and negatively associated with well-being (*r* = −0.59, *p* < 0.001) and perceived social support (*r* = −0.37, *p* < 0.001). However, no comparable significant associations were observed among younger men.

Among middle-aged women, Change in suicidal ideation was significantly associated with well-being (*r* = −0.21, *p* = 0.023) and perceived social support (*r* = −0.21, *p* = 0.026). Among middle-aged men, Change in suicidal ideation was significantly associated with depressive symptoms (*r* = 0.41, *p* < 0.001) and perceived social support (*r* = −0.29, *p* = 0.003). The associations between suicidal ideation and other constructs among middle-aged women and middle-aged men were relatively weaker than those among younger women.

**Figure 2:**
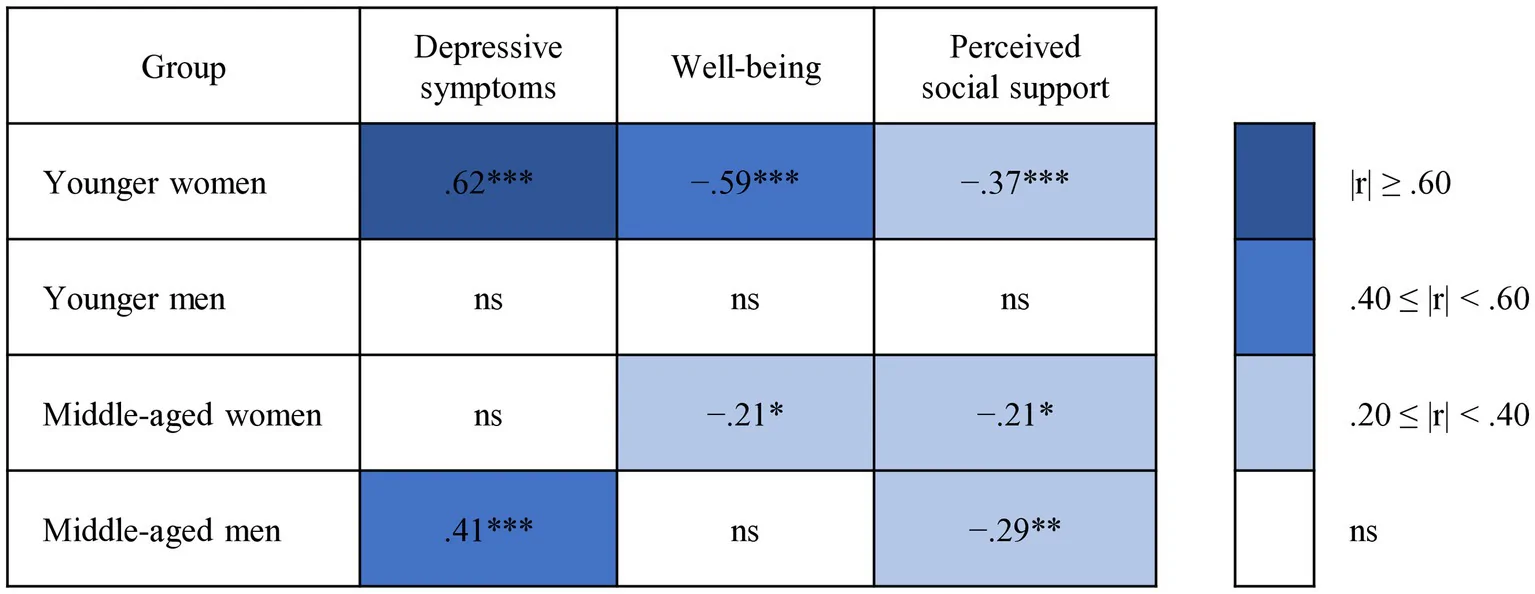
Summary of Change–Change correlations between suicidal ideation and depressive symptoms, well-being, and perceived social support by age-by-sex group. Values represent correlations between latent change factors. Color intensity indicates the absolute magnitude of statistically significant correlations. ns, not significant. ^*^p < 0.05, ^**^p < 0.01, ^***^p < 0.001. Correlations do not indicate causal direction.

To directly test whether these apparent sex differences were statistically supported, we conducted supplementary nested model comparisons within each age group. Among younger participants, all three focal Change–Change associations—between change in suicidal ideation and changes in depressive symptoms, well-being, and perceived social support—differed significantly between women and men. In contrast, among middle-aged participants, none of these sex differences reached statistical significance. These results indicate that sex differences in the short-term co-change pattern were statistically supported among younger participants, but not among middle-aged participants. Detailed results of these nested model comparisons are presented in Supplementary Table S2. Figure 2 summarizes the Change–Change correlations between suicidal ideation and the three study constructs across the four age-by-sex groups.

### 3.6 Sensitivity analyses

As a supplementary sensitivity analysis, we also examined whether observed change in suicidal ideation (T2–T1) was associated with age and observed changes in depressive symptoms, well-being, and perceived social support within each age-by-sex group. The pattern of results was partly consistent with, but not identical to, the latent change model. Age was a significant positive predictor only among younger women. No significant predictors were observed among younger men. Among younger women, increases in depressive symptoms, decreases in well-being, and higher age were associated with greater increases in suicidal ideation. Among middle-aged men, increases in depressive symptoms were associated with greater increases in suicidal ideation, whereas no predictors reached significance among middle-aged women. Detailed results are presented in Supplementary Table S3.

Sensitivity analyses excluding item 9 of the PHQ-9 yielded results in the same direction as the main analyses, indicating that the overall pattern of findings was robust to potential item overlap between depressive symptoms and suicidal ideation.

## 4 Discussion

This study examines perceived social support, depressive symptoms, and well-being as suicide-related indicators. We examined whether changes in these variables covary with changes in suicidal ideation and whether these associations vary in strength by age and sex.

Among young people, women showed higher suicidal ideation and depressive symptoms and lower well-being than men, whereas there were no sex differences in perceived social support. In middle adulthood, mental health did not differ by sex, while perceived social support was higher among women. Although higher levels of suicidal ideation among women have been reported across many age groups (Boyd et al., 2015), these findings showed this pattern only among young people.

### 4.1 Suicidal ideation and perceived social support

In this study, associations between Change in suicidal ideation and Change in perceived social support were significant in all groups except younger men. Although perceived social support was concurrently related to depressive symptoms and well-being, Change in perceived social support was also significantly associated with Change in suicidal ideation. The association was strongest among younger women, and the magnitudes were generally comparable between middle-aged men and women.

Among younger women, perceived social support was associated with suicidal ideation, whereas no such association was observed among younger men. Previous research indicates that the protective effect of social support is stronger for younger women than younger men (Johansen et al., 2021). In this study, most young people were unmarried, and the measured social support likely reflected relationships primarily with parents, siblings, and friends. Regarding men in early adulthood, such support may not necessarily buffer suicidal ideation, whereas disruptions in spousal relationships, such as divorce or conflict with a partner, may increase suicide risk (Kposowa, 2000). Therefore, among younger men, the overall social support assessed in this study may have had a relatively weaker association with suicidal ideation. At the same time, the meaning of perceived social support may not have been identical across age groups. Because younger participants were mostly unmarried, perceived support may have primarily reflected family- and friend-based support, whereas among middle-aged participants it may have more strongly reflected support from spouses and workplace relationships. In addition, because the PERMA-Profiler includes a relationships component, perceived social support and well-being may partially overlap, particularly in groups for whom interpersonal connectedness is central to daily functioning. However, among younger men, prevailing social norms may discourage the use of available support in mental health contexts (Sheikh et al., 2025). This may be particularly relevant for interpreting the weaker association observed among younger men. Future research should therefore also examine how other dimensions of social support—such as support seeking (e.g., the Actual Help-Seeking Questionnaire; Wilson et al., 2005) and received support (e.g., the Inventory of Socially Supportive Behaviors; Barrera et al., 1981)—are associated with suicidal ideation among younger men.

In middle adulthood, social support was associated with suicidal ideation for both women and men. Because participants in middle adulthood were more likely to be married and employed, the measured social support may have strongly reflected relationships with spouses and the workplace. Differences in marital status and the presence of a spouse influence suicide risk, and marital status (e.g., being unmarried, widowed, divorced, or separated) and the quality of spousal relationships are closely associated with suicide mortality risk, particularly among men (Denney et al., 2009; Fukuchi et al., 2013; Kposowa, 2000). Additionally, among workers, lower workplace social capital is associated with increased risk of suicidal ideation for both women and men (Hori et al., 2019). Collectively, these findings suggest that in middle adulthood, social support derived from spouses and the workplace may become more directly linked to mental health and suicidal ideation.

### 4.2 Suicidal ideation and the two-factor model of mental health

Associations between suicidal ideation and the two dimensions of mental health—well-being and depressive symptoms—differed slightly by sex and age. Among younger women, both well-being and depressive symptoms were associated with suicidal ideation, and the strengths of the associations were similar for depressive symptoms, well-being, and suicidal ideation; in contrast, these associations were unclear among younger men. This pattern is consistent with previous evidence that the association between depressive symptoms and suicidal ideation is more pronounced among women in young adulthood (Allison et al., 2001; Ibrahim et al., 2017) and that lower life satisfaction is more strongly linked to suicidal ideation among women (Hossain et al., 2016; Lu et al., 2020). Previous studies have similarly suggested that depressive symptoms and positive mental health indicators may be less strongly associated with suicidal ideation among young men than among young women (Hossain et al., 2016; Ibrahim et al., 2017; Lamis and Lester, 2013). These findings have been interpreted as suggesting that other factors, including alcohol-related problems, coping and problem-solving patterns, and reluctance to express distress or seek support, may be relatively more important among young men. However, because these previous studies were cross-sectional, they cannot establish temporal relationships and do not directly explain the absence of short-term Change–Change associations in the present study. The present findings may therefore indicate that short-term changes in suicidal ideation among younger men are associated with factors not assessed in this study, but this possibility requires further longitudinal investigation.

In middle adulthood, depressive symptoms were associated with suicidal ideation among men, whereas well-being was associated with suicidal ideation among women, and sex differences were less pronounced than among young people. This interpretation was further supported by direct between-group tests, which showed significant sex differences in the key Change–Change associations among younger participants, but not among middle-aged participants. Accordingly, the present findings suggest that sex-specific short-term co-change patterns may be more pronounced in younger adulthood than in middle adulthood. Sex differences in the associations between suicidal ideation and mental health (depressive symptoms and well-being) may be present in young adulthood but may diminish from middle adulthood onward. Additionally, the negative association between well-being and suicidal ideation was observed among women in both young and middle adulthood, suggesting that well-being may be a consistent protective factor across the female life span.

Recent studies suggest that sex differences in suicidal ideation may reflect different mechanisms across populations and contexts. Neuroimaging studies in major depressive disorder report sex differences in brain structural correlates of suicidal ideation (Liu et al., 2025; Xia et al., 2025). Population-based studies also indicate sex-specific patterns in how depressive and anxiety symptoms relate to suicidal ideation, including among older adults (Li et al., 2025; Rashidi et al., 2024). Among adolescents, the association between coping and suicidal ideation may differ by sex and age (Gómez-Tabares and Restrepo, 2025). These findings provide a broader context for interpreting the present age-by-sex differences in short-term changes in suicidal ideation and related psychosocial factors.

### 4.3 Implications and limitations

These findings suggest that sex differences in factors associated with suicidal ideation may vary by life stage. These results may also have implications for prevention and support strategies. For younger women, multi-component approaches targeting depressive symptoms, well-being, and psychosocial support may be particularly relevant, consistent with evidence that psychosocial interventions can reduce suicidal ideation in young people (Robinson et al., 2018). For younger men, broader strategies that facilitate engagement with support and help-seeking may warrant further study. Among middle-aged men, depression screening and access to appropriate care, together with support in workplace or community contexts, may be important considerations. Among middle-aged women, interventions that strengthen connectedness and community-based support may be especially relevant (World Health Organization, 2019). In practice, these patterns may help guide the domains prioritized in assessment and support planning: depressive symptoms, positive mental health, and social connectedness may need to be considered together rather than focusing on depressive symptoms alone, while broader assessment may be particularly important for younger men. The significant Change–Change correlations ranged in magnitude from |r| = 0.21 to 0.62, indicating that the strength of group-level co-change varied across age-by-sex groups. However, these correlations do not establish clinically meaningful change at the individual level or demonstrate intervention effects.

This study examines changes in suicidal ideation over a one-month interval; future research should examine whether similar longitudinal relationships are observed over longer periods or at shorter intervals. In addition, attrition bias should be considered, as respondents who dropped out after T1 were younger and reported higher depressive symptoms at baseline than those who completed both waves. Consequently, the analytic sample may underrepresent younger individuals with higher levels of depressive symptoms, and the observed associations may not fully reflect the relationships among suicidal ideation, mental health, and perceived social support in this population. Suicidal ideation can fluctuate even within a day (Gee et al., 2020; Rabasco and Sheehan, 2022). Future research should examine whether increases in connectedness with others and well-being are associated with short-term reductions in elevated suicidal ideation.

Another limitation concerns potential content overlap between depressive symptoms and suicidal ideation, because item 9 of the PHQ-9 includes death- and self-harm-related thoughts. However, sensitivity analyses excluding this item yielded results in the same direction as the main analyses, suggesting that item overlap alone does not explain the observed associations.

In addition, a supplementary sensitivity analysis treating age as a continuous variable showed that age was significantly associated with observed change in suicidal ideation only among younger women. Although this finding suggests that the main results were not solely driven by the predefined age cut-points, it also indicates that some within-group age heterogeneity may remain, particularly among younger women. These supplementary analyses should be interpreted cautiously, because regression using observed difference scores is not equivalent to the latent change model used in the main analyses and does not account for measurement error in the same way.

Although the findings of this study may inform future interventions aimed at reducing suicidal ideation, the present design does not allow conclusions about intervention effects or causal relationships. Having suicidal ideation does not necessarily indicate the occurrence of suicidal behavior or death by suicide; therefore, this study cannot conclude that enhancing perceived social support or improving well-being is effective for preventing suicidal behavior. However, changes in suicidal ideation covary with changes in psychological distress (Troister et al., 2013). Moreover, reducing suicidal ideation may help individuals experience lower distress. Particularly, frequent suicidal ideation during adolescence and recent suicidal ideation predict suicide attempt(s) several years later even after controlling for other factors (Miranda et al., 2014). Therefore, although the present findings should not be interpreted as evidence of prevention effects, reductions in suicidal ideation may still be relevant to alleviating distress and may have implications for future suicide prevention.

Another limitation is that perceived social support and well-being may have partially overlapping interpersonal content, and the functional meaning of perceived support may have differed across age groups depending on marital, family, and occupational roles.

Because change was assessed over only two time points separated by 1 month, the findings should be interpreted as evidence of short-term co-change among the constructs rather than as evidence of stable or long-term developmental change. In addition, all measures were based on self-report, which may have introduced reporting bias and shared method variance. External validity is also limited. Because participants were recruited from an online panel using quota sampling, and participated voluntarily on a first-come, first-served basis until the predetermined quotas were filled, the sample may overrepresent panel members who responded promptly. Therefore, the findings may not generalize directly to the broader Japanese population.

Because multiple supplementary group comparisons were conducted, these results should be interpreted cautiously and considered supportive rather than definitive.

## 5 Conclusion

This study identified age-related sex differences in short-term Change–Change associations between suicidal ideation and perceived social support, well-being, and depressive symptoms over a one-month interval. Perceived social support and well-being were consistently associated with lower short-term change in suicidal ideation among women, whereas younger men showed no significant Change–Change associations, suggesting that correlates of suicidal ideation may differ across age-by-sex groups. Future research using more than two waves and assessing additional risk and protective factors is warranted, particularly to elucidate mechanisms relevant to younger men.

## Data Availability

The datasets presented in this study can be found in online repositories. The names of the repository/repositories and accession number(s) can be found below: The de-identified data for this study are publicly available on the Open Science Framework (OSF); Accession number: 10.17605/OSF.IO/WVBAX; Repository URL: https://doi.org/10.17605/OSF.IO/WVBAX.
