# Supplementary material for: Age-related sex differences in factors associated with suicidal ideation: a short-term longitudinal study
Source: Front Psychol. 2026 Aug 19;17:1813220. doi: 10.3389/fpsyg.2026.1813220 (PMC13533977; doi:10.3389/fpsyg.2026.1813220)
Supplement: Supplementary file 1 [file Table_1.DOCX]

**Supplementary Table S1**. Comparison of completers and dropouts on baseline characteristics

|  | Completers  (*n* = 1,078) | | Dropouts  (*n* = 447) | | Test statistics | *p* |
| --- | --- | --- | --- | --- | --- | --- |
| Sex, n (%) |  |  |  |  | 0.01 | .934 |
| Men | 521 | (48.3) | 215 | (48.1) |  |  |
| Women | 557 | (51.7) | 232 | (51.9) |  |  |
| Age, mean (*SD*) | 33.18 | (10.48) | 25.19 | (9.60) | 14.39 | <.001 |
| T1 Perceived social support, mean (*SD*) | 53.11 | (19.63) | 53.96 | (19.77) | 0.77 | .442 |
| T1 Depressive Symptoms, mean (*SD*) | 6.37 | (6.61) | 7.37 | (6.84) | 2.62 | .009 |
| T1 Well-being, mean (*SD*) | 4.99 | (2.33) | 5.11 | (2.20) | 1.00 | .317 |
| T1 Suicidal ideation, mean (*SD*) | 8.39 | (3.06) | 8.36 | (3.08) | 0.15 | .885 |

*Note.* Test statistics represent independent-samples *t* tests for continuous variables and a chi-square test for sex distribution.
