# Supplementary material for: Age-related sex differences in factors associated with suicidal ideation: a short-term longitudinal study
Source: Front Psychol. 2026 Aug 19;17:1813220. doi: 10.3389/fpsyg.2026.1813220 (PMC13533977; doi:10.3389/fpsyg.2026.1813220)
Supplement: Supplementary file 2 [file Table_2.DOCX]

**Supplementary Table S2**. Nested multi-group comparisons of key Change–Change associations across sex within each age group

| Comparison | Constrained association | Unconstrained *χ²*(*df*) | Constrained *χ²*(*df*) | Δ*χ²* | Δ*df* | *p* |
| --- | --- | --- | --- | --- | --- | --- |
| Younger women vs younger men | SI Change ↔  PSS Change | 4100.62(1952) | 4109.73(1953) | 9.11 | 1 | .003 |
| Younger women vs younger men | SI Change ↔  DEP Change | 4100.62(1952) | 4119.48(1953) | 18.85 | 1 | .000 |
| Younger women vs younger men | SI Change ↔  WB Change | 4100.62(1952) | 4118.38(1953) | 17.76 | 1 | .000 |
| Middle-aged women vs Middle-aged men | SI Change ↔  PSS Change | 4172.36(1952) | 4173.48(1953) | 1.12 | 1 | .290 |
| Middle-aged women vs Middle-aged men | SI Change ↔  DEP Change | 4172.36(1952) | 4176.07(1953) | 3.71 | 1 | .054 |
| Middle-aged women vs Middle-aged men | SI Change ↔  WB Change | 4172.36(1952) | 4172.68(1953) | 0.32 | 1 | .573 |

*Note.* Each test compares an unconstrained model with a model constraining the focal Change–Change association to be equal across the two specified groups. A significant chi-square difference indicates that the association differs significantly between groups. SI = suicidal ideation; PSS = perceived social support; DEP = depressive symptoms; WB = well-being.
