# Supplementary material for: Age-related sex differences in factors associated with suicidal ideation: a short-term longitudinal study
Source: Front Psychol. 2026 Aug 19;17:1813220. doi: 10.3389/fpsyg.2026.1813220 (PMC13533977; doi:10.3389/fpsyg.2026.1813220)
Supplement: Supplementary file 3 [file Table_3.DOCX]

**Supplementary Table S3**. Group-specific sensitivity analyses predicting observed change in suicidal ideation (T2–T1)

|  | Younger Women | | Younger Men | | Middle-aged Women | | Middle-aged  Men | |
| --- | --- | --- | --- | --- | --- | --- | --- | --- |
|  | *B (SE)* | *p* | *B (SE)* | *p* | *B (SE)* | *p* | *B (SE)* | *p* |
| Age | .05 (.02) | .027 | -.01 (.02) | .445 | -.01 (.01) | .436 | .00 (.01) | .810 |
| Change in PSS | -.01 (.01) | .533 | .01 (.01) | .454 | -.01 (.01) | .136 | -.01 (.01) | .079 |
| Change in DEP | .10 (.02) | <.001 | .02 (.03) | .431 | .03 (.02) | .216 | .07 (.02) | <.001 |
| Change in WB | -.33 (.09) | <.001 | -.11 (.08) | .180 | -.09 (.07) | .170 | -.05 (.06) | .448 |
| *R²* | .24 |  | .02 |  | .03 |  | .06 |  |

*Note.* The dependent variable was observed change in suicidal ideation (T2–T1). Predictors were entered simultaneously within each age-by-sex group. Positive coefficients indicate greater increases in suicidal ideation over time. PSS = perceived social support; DEP = depressive symptoms; WB = well-being.
