# Supplementary material for: Age-related sex differences in factors associated with suicidal ideation: a short-term longitudinal study
Source: Front Psychol. 2026 Aug 19;17:1813220. doi: 10.3389/fpsyg.2026.1813220 (PMC13533977; doi:10.3389/fpsyg.2026.1813220)
Supplement: Supplementary file 4 [file Table_4.DOCX]

| Construct | Model | *χ²* | *df* | *p* | CFI | RMSEA | SRMR | ΔCFI | ΔRMSEA | ΔSRMR |
| --- | --- | --- | --- | --- | --- | --- | --- | --- | --- | --- |
| PSS | 0 | 25.37 | 20 | .19 | 0.999 | 0.016 | 0.008 |  |  |  |
| PSS | 1 | 44.85 | 28 | .02 | 0.998 | 0.024 | 0.008 | -0.001 | 0.008 | 0.000 |
| PSS | 2 | 53.00 | 40 | .08 | 0.998 | 0.017 | 0.008 | 0.000 | -0.007 | 0.000 |
| PSS | 3 | 58.31 | 46 | .11 | 0.999 | 0.016 | 0.009 | 0.001 | -0.001 | 0.001 |
| PSS | 4 | 116.03 | 55 | <.001 | 0.993 | 0.032 | 0.010 | -0.006 | 0.016 | 0.001 |
| DEP | 0 | 1493.62 | 500 | <.001 | 0.935 | 0.043 | 0.057 |  |  |  |
| DEP | 1 | 1532.50 | 532 | <.001 | 0.935 | 0.042 | 0.063 | 0.000 | -0.001 | 0.006 |
| DEP | 2 | 1588.91 | 568 | <.001 | 0.933 | 0.041 | 0.063 | -0.002 | -0.001 | 0.000 |
| DEP | 3 | 1616.85 | 592 | <.001 | 0.933 | 0.040 | 0.065 | 0.000 | -0.001 | 0.002 |
| DEP | 4 | 1725.57 | 619 | <.001 | 0.928 | 0.041 | 0.067 | -0.005 | 0.001 | 0.002 |
| WB | 0 | 569.62 | 116 | <.001 | 0.971 | 0.060 | 0.028 |  |  |  |
| WB | 1 | 586.49 | 132 | <.001 | 0.971 | 0.057 | 0.028 | 0.000 | -0.003 | 0.000 |
| WB | 2 | 623.79 | 152 | <.001 | 0.969 | 0.054 | 0.028 | -0.002 | -0.003 | 0.000 |
| WB | 3 | 631.20 | 164 | <.001 | 0.970 | 0.052 | 0.026 | 0.001 | -0.002 | -0.002 |
| WB | 4 | 699.60 | 179 | <.001 | 0.966 | 0.052 | 0.032 | -0.004 | 0.000 | 0.006 |
| SI | 0 | 503.67 | 188 | <.001 | 0.969 | 0.040 | 0.039 |  |  |  |
| SI | 1 | 545.51 | 208 | <.001 | 0.967 | 0.039 | 0.039 | -0.002 | -0.001 | 0.000 |
| SI | 2 | 609.51 | 232 | <.001 | 0.963 | 0.039 | 0.039 | -0.004 | 0.000 | 0.000 |
| SI | 3 | 658.39 | 247 | <.001 | 0.960 | 0.039 | 0.049 | -0.003 | 0.000 | 0.011 |
| SI | 4 | 696.86 | 265 | <.001 | 0.958 | 0.039 | 0.051 | -0.002 | 0.000 | 0.001 |

**Supplementary Table S4.** Longitudinal and between-group measurement invariance analyses for the study constructs

*Note:* Δ values were calculated as the fit index for the more constrained model minus that for the immediately preceding model. Thus, negative ΔCFI values and positive ΔRMSEA and ΔSRMR values indicate deterioration in model fit. PSS = perceived social support; DEP = depressive symptoms; WB = well-being; SI = suicidal ideation. Model 0 = configural model; Model 1 = longitudinal metric invariance; Model 2 = longitudinal scalar invariance; Model 3 = between-group metric invariance; Model 4 = between-group scalar invariance.
